# Is a Four-Week Ketogenic Diet an Effective Nutritional Strategy in CrossFit-Trained Female and Male Athletes?

**DOI:** 10.3390/nu13030864

**Published:** 2021-03-06

**Authors:** Krzysztof Durkalec-Michalski, Paulina M. Nowaczyk, Natalia Główka, Anna Ziobrowska, Tomasz Podgórski

**Affiliations:** 1 Department of Sports Dietetics, Poznan University of Physical Education, 61-871 Poznań, Poland; 2 Department of Human Nutrition and Dietetics, Poznan University of Life Sciences, 60-624 Poznań, Poland; paulina.nowaczyk@up.poznan.pl (P.M.N.); natalia.glowka@up.poznan.pl (N.G.); anna.w.ziobrowska@gmail.com (A.Z.); 3 Department of Physiology and Biochemistry, Poznan University of Physical Education, 61-871 Poznań, Poland; podgorski@awf.poznan.pl

**Keywords:** low-carbohydrate, high-fat diet, high-intensity functional training, sports nutrition, nutritional support, exercise performance, physical capacity

## Abstract

This single-arm interventional study examined the effect of a 4-week ketogenic diet (KD) on aerobic capacity and discipline-specific performance in female (*n* = 11) and male (*n* = 11) CrossFit-trained athletes. The participants performed incremental cycling (ICT) and Fight Gone Bad (FGB) tests after consuming a customary diet and a KD. Pre- and post-ICT exercise blood samples were also analysed. Consuming a KD had a slight impact on aerobic capacity and no relevant effect on CrossFit-specific performance. In females, consuming a KD led to an 10.4% decrease in peak oxygen uptake during the ICT (*p* = 0.027) and resulted in certain alterations in haematological parameters (haemoglobin (HGB), mean corpuscular HGB, and mean corpuscular HGB concentration). Furthermore, in males, alanine aminotransferase activity increased with a simultaneous improvement in the post-ICT blood acid–base balance after consuming a KD. The pre-exercise bilirubin concentration was also elevated in the entire group after consuming a KD. In conclusion, female CrossFit-trained athletes seem to be prone to aerobic performance decrements and increased risk of developing haematological disturbances when consuming a KD. In males who consumed a KD, there was an undesirable alanine aminotransferase elevation and a small tendency towards improved acid–base status. Moreover, consuming a KD had no effect on discipline-specific performance in CrossFit-trained athletes.

## 1. Introduction

Providing muscles with substrates to fuel the energy needs of exercise is one of the most important goals in sport nutrition. Adequate nutrient intake and endogenous stores are crucial to achieve good performance, enhance physical capacity, ensure proper nutritional status, and provide optimal exercise-induced adaptation [1]. Energy sources for the human body are carbohydrates (exogenous or stored in the form of glycogen), fats (free fatty acids or stored in the form of body fat) [1] and, alternatively, ketone bodies (KBs) [2]. Carbohydrates are the key energy substrate in sport, especially in high-intensity efforts [1]. Furthermore, due to athletes’ physiological demands, recommendations released by leading scientific societies, including the International Society of Sports Nutrition, the American College of Sports Medicine, and the Australian Institute of Sport, recommend optimal carbohydrate intake before, during, and after training or competition [3,4,5]. However, a serious and frequently reported problem in the literature is the limited carbohydrate availability in athletes, or even their deliberate intention to limit them (e.g., during weight loss or adipose tissue reduction). This phenomenon may become a limiting factor in terms of performance in prolonged submaximal or intermittent high-intensity training [1,6]. Therefore, to improve the metabolic efficiency of muscles, attempts have been made to adapt alternative directions of nutritional strategies.

In recent years, researchers have mainly focused on periodisation and manipulation of carbohydrates to decrease the rate of their utilisation and simultaneously increase fat metabolism during and after exercises. A ketogenic diet (KD), which is a low-carbohydrate, high-fat diet, is assumed to induce such alterations in exercise metabolism [7,8].

There have been numerous KD modifications, but it was originally designed with a 4:1 lipid-to-nonlipid ratio, where 80% of total energy intake is provided from fat, 15% from protein and 5% from carbohydrates [7,9]. At first, KD was intended as a treatment for epilepsy and has been prescribed for this disease for almost 100 years [9], whereas the first studies on the metabolic aspects of a KD were conducted in the late 1960s [7]. A KD can induce a metabolic state called ‘ketosis’ [7]. In this state, serum KBs are elevated (0.5–3.0 mmol·L^−1^) and their cellular oxidation is enhanced [9]. A KD may also stimulate fat oxidation and promote fat loss [9]. In contrast to ketosis, a pathological condition called ‘ketoacidosis’ is characterised by a decreased pH in arteries (from 7.30 to 7.20) and elevated serum ketone levels (3.8–25 mmol·L^−1^) [9,10]. Furthermore, low availability of carbohydrates supposedly mobilises fatty acids from adipose tissue, which is a phenomenon that can promote weight loss [11,12,13,14]. Moreover, low carbohydrate intake is associated with lower cholesterol and insulin concentrations, improved blood lipid profiles, and reduced cardiovascular disease risk factors [12,13,14]. A KD is also a promising therapy for certain types of cancer (e.g., human gastric adenocarcinoma or squamous cell carcinoma) [15,16].

In sport and physical activity, exercise intensity and duration have an influence on the contribution of fatty acids to oxidative metabolism. Specifically, exercise of a longer duration and lower intensity increase this contribution. Thus, a KD may be beneficial for endurance training [9]. The main mechanism thought to be responsible for the impact of consuming a KD is upregulation of the release, transport, uptake, and utilisation of fat in the muscle [6]. Increased hepatic KBs production and maximising the rate of fat oxidation may provide additional energy substrates for muscles and the central nervous system [17,18]. Nevertheless, the contribution of KBs as an exercise energy substrate is affected by several factors, including the protocol used to achieve ketosis or training status [19,20]. Presumably, there are also other mechanisms, e.g., molecular upregulation. In an animal model, a KD improved mitochondrial biogenesis and bioenergetics via the peroxisome proliferator-activated receptor γ coactivator 1–sirtuin 3–uncoupling protein 2 (PGC1α–SIRT3–UCP2) axis [21]. Furthermore, reduced muscle glycogen and exogenous glucose availability enhance both AMP-activated protein kinase (AMPK) and p38 mitogen-activated protein kinase (p38MAPK) phosphorylation. This results in activation and translocation of PGC1α and p53 kinase to mitochondria and the nucleus. In this way, low carbohydrate availability may influence training adaptations by modulating cell signalling pathways that are sensitive to nutrients, for example, enhancing mitochondrial adaptations to promote aerobic capacity potential [22,23,24,25]. In addition, physical efforts under low carbohydrate availability may support adipose tissue mobilisation, lipolysis efficiency (e.g., via increased circulating epinephrine concentrations), and fat oxidation [8,22]. By contrast, a high-carbohydrate diet/intake suppresses fat utilisation and elevates carbohydrate oxidation or downregulates lipolysis as well as reducing both AMPK and p38MAPK activity during exercise [22,26]. Finally, despite these adaptive responses, carbohydrate deficiency seems to be one of the most important nutritional triggers that impairs exercise performance [22]. It is also still unclear whether high-fat diets impair muscle protein synthesis [27] by suppressing the mechanistic target of rapamycin (mTOR) and 70 kDa ribosomal protein S6 kinase (p70S6K) signalling pathway (despite leucine-rich protein intake) [22].

Several studies have reported the effects of consuming a KD on athletes, but they did not record any promising ergogenic results [28,29,30,31]. Recent studies performed on athletes have reported significant changes in body composition and fat mass content [17,32,33]. However, a KD seems ineffective in improving exercise performance, time to exhaustion [17], maximal oxygen uptake [8], or endurance cycling performance [33]. Other studies evaluating the effects of a KD on anaerobic performance have not revealed significant results in strength or power measures [34,35]. In addition, performance at higher intensities may even be impaired. Currently, due to potential negative effects and inconsistent scientific evidence, a KD does not seem to be recommended for athletes [9]. Nevertheless, alternative dietary and supplementation strategies are becoming more attractive to sport communities at the competitive and recreational level [9,36]. Therefore, there is a need for further studies on KD use in athletes to assess its potential impact in sport. 

In our previous work, it was found that in CrossFit-trained athletes, a 4-week KD shifted macronutrient utilisation (in favour of fat utilisation). The effect was more pronounced in males compared with females [8]. As a follow-up, it was aimed to determine whether the mentioned alterations in exercise metabolism would impact aerobic adaptation, blood biomarker concentration/activity or discipline-specific performance. It was hypothesised that a 4-week KD does not improve aerobic capacity during the incremental cycling test (ICT) or CrossFit-specific performance (Fight Gone Bad (FGB) exercises). Our secondary hypothesis was that a KD does not induce changes in biochemical and haematological blood markers or blood acid–base balance. A single-arm nutritional intervention trial was conducted in a group of CrossFit-trained athletes (11 females and 11 males) who consumed a KD for 4 weeks. 

## 2. Materials and Methods

### 2.1. Participants

**Figure 1:**
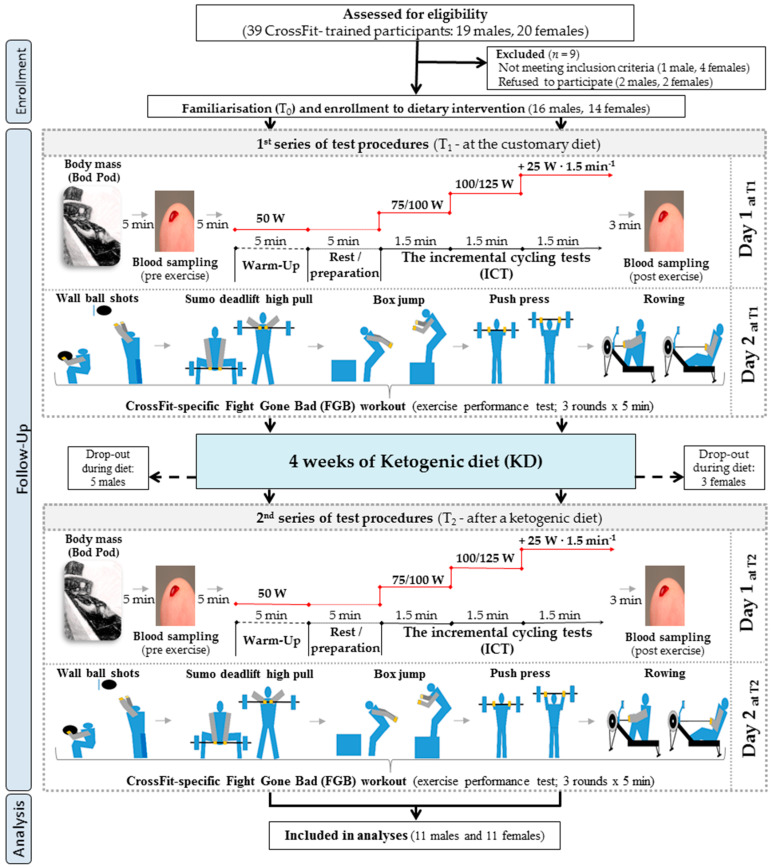
A flow chart of the study design.

Thirty participants were initially enrolled in this study. Twenty-two of them (11 females and 11 males) completed the study protocol and were included in the analyses (Figure 1 and Table 1). All participants were recreational CrossFit athletes, members of four CrossFit clubs in Poznań, Poland (Caffeine Barbell, Hangar, Reebok CrossFit Poznań, and Rankor Athletics). Inclusion criteria were age between 18 and 40 years, a valid and up-to-date medical certificate of the athlete’s ability to practice sports, at least 2 years of CrossFit training experience, and at least four CrossFit trainings per week. Both males and females were included because of the equal participation of both sexes in CrossFit training and to assess whether a KD has sex-specific impacts. Exclusion criteria were smoking, drug and dietary supplement usage, alcohol consumption (>1–2 drinks/week), consuming a special diet within 3 weeks of the study’s commencement, and pregnancy or menstrual cycle disorders.

The study protocol was reviewed and approved by the local institutional review board (Bioethics Committee at Poznań University of Medical Sciences, reference numbers: 681/16 and 683/16, 10 November 2016). Written informed consent was obtained from all participants before the study began. All procedures were conducted in accordance with the ethical standards of the 1964 Declaration of Helsinki. The trial was conducted from January to December 2017. This trial was registered at ClinicalTrials.gov (Clinical Trial Identification Number: NCT03665948; https://www.clinicaltrials.gov/ct2/show/NCT03665948, accessed on 2 March 2021). The study was registered retrospectively because registration was not required when the study enrolment started. The authors confirm that all ongoing and related trials for this intervention are registered.

### 2.2. Study Design, Protocol, and Visits

The entire study protocol and diet characteristics are thoroughly described in our previous paper [8]. The study protocol included three visits (T_0_, T_1_, and T_2_) to the laboratory (Figure 1). The CrossFit participants were very familiar with the CrossFit-specific exercise tests. Furthermore, the participants were familiarised with the entire study protocol, such as the ICT, anthropometric measurements, food recording method, and physical activity questionnaire, during the first visit (T_0_).

The KD diet was individualised to ensure weight maintenance (assessed using a Bod Pod^®^ analyser; Cosmed, Rome, Italy) throughout the dietary intervention, and diets were energetically normalised (based on estimated energy expenditure via heart rate (HR) monitoring using a Polar RS-400, Vantaa, Finland, as described previously [8,37,38]). The main dietary assumptions concerned the intake of ≥75% daily energy from fat, 1.7 g of protein per kilogram of body mass, and up to 5% energy from carbohydrates [8]. Participants following their customary diet (CD) were obligated to record all ingested food and beverages in a food diary during a 14-day run-in phase, after which the first laboratory study visit was conducted (T_1_). The day after T_1_, the same participants started following 10-day KD menus for 4 weeks and recorded their food intake using a food diary and an electronic kitchen scale. After the KD intervention, the second series of test procedures (T_2_) was conducted. The subjects were asked to maintain a similar training load and the same physical activity level throughout the study. Final diet intake is presented in Supplementary Table S1. Furthermore, the KD compliance had been confirmed by ongoing monitoring of the beta-hydroxybutyrate (βHB) concentration in blood and the KB level in urine (urine ketones were tested during T_1_ and T_2_ study visits, and once weekly during KD) [8].

### 2.3. Exercise Tests

#### 2.3.1. ICT

The ICT was performed at the Exercise Tests Laboratory at the Institute of Human Nutrition and Dietetics under standardised conditions in the morning and on a Kettler X1 cycloergometer (Kettler, Ense-Parsit, Germany) according to our previously published protocol [8]. Three hours before the T_1_ and T_2_ visits, the participants consumed a standardised meal corresponding to their habitual food intake or a meal corresponding to the KD menu, respectively. Cardiorespiratory indices were measured (breath by breath) using the Quark CPET ergospirometer (Cosmed, Rome, Italy). The analysed variables include peak oxygen uptake (VO_2peak_); maximal workload (W_max_); time to exhaustion (T_exh_); maximum heart rate (HR_max_); and gas exchange threshold (GET), including time to GET (T_GET_), oxygen uptake at GET (VO_2GET_), percentage of maximum oxygen uptake at GET (% VO_2maxGET_), workload at GET (W_GET_), and heart rate at GET (HR_GET_). The VO_2peak_ was considered to be the moment when the individual oxygen uptake (VO_2_) recorded during the ICT reached the highest point. The VO_2max_ was defined as the maximum minute VO_2_ when a workload increase stopped generating further increases in VO_2_ and HR and/or the athlete refused to continue exercising [8,37]. To determine the GET during the ICT, the V-slope method was applied based on an analysis of the linear regression for the curve of increasing CO_2_ exhalation in comparison to the curve of increasing O_2_ uptake [37,39,40]. During the entire test, participants were encouraged by verbal motivation.

#### 2.3.2. CrossFit-Specific Physical Performance Test

The FGB workout was performed according to the protocol from our previous study [41]. The entire test lasted for 17 min (3 rounds × 5 min and 2 breaks × 1 min, between the first and second and the second and third rounds). Each single 5 min round consisted of five exercises: (a) wall ball shots, (b) sumo deadlift high pulls, (c) box jumps, (d) push presses, and (e) rowing. CrossFitters were obligated to complete as many repetitions as possible at each FGB station prior to moving to the next station. The FGB test was supervised by experienced CrossFit coaches to allow an accurate count of all properly executed repetitions. For each valid repetition, a participant needed to complete a full range of motion required for a specific exercise. Each FGB test was performed in the afternoon on the day after the ICT. It was performed in the natural condition in CrossFit boxes where the participants usually trained. Moreover, during exercise tests, the researchers and coaches provided verbal encouragement.

### 2.4. Blood Collection and Sample Analysis

#### 2.4.1. Blood Sampling

Capillary blood samples were obtained in the resting state (before pre-ICT warm-up) and 3 min post-ICT exercises. All blood samples were obtained in a seated upright position from the nondominant hand using a Medlance^®^ Red lancet-spike (HTL-Zone, Berlin, Germany) with a 1.5 mm blade and 2.0 mm penetration depth.

#### 2.4.2. Blood Sample Analysis

For blood collected in heparinised capillary tubes (65 μL), the pH, concentration of bicarbonate (HCO_3_^−^) and hydrogen (H^+^) ions, standard base excess (BE), anion gap, lactate, glucose (GLU), bilirubin (BIL), and plasma osmolality were determined using a gasometric analyser (ABL90 FLEX, Radiometer, Copenhagen, Denmark). Additionally, 300 µL of capillary blood was collected in a Microvette^®^ CB 300 tube (Sarstedt, Nümbrect, Germany) containing EDTA dipotassium salt as an anticoagulant for haematological measurement using a 20-parametric automated Mythic^®^18 haematology analyser (Orphée, Switzerland). Another 300 µL of capillary blood was collected in a Microvette^®^ CB 300 Z tube (Sarstedt, Nümbrect, Germany) with a clotting activator, in which the concentrations of total testosterone (DRG MedTek, Warsaw, Poland; Cat No. EIA-1559) and cortisol (DRG MedTek, Warsaw, Poland; Cat No. EIA-1887) were determined using commercially available enzyme-linked immunosorbent assay (ELISA) kits. The absorbance readings were taken on a multi-detection microplate ELISA reader (Synergy 2 SIAFRT, BioTek, Vermont, USA). The urea concentration (Cormay, Łomianki, Poland; Cat No. 2-206), and the alanine aminotransferase (AlAT; EC 2.6.1.2; Cormay, Łomianki, Poland; Cat No. 1-221), aspartate aminotransferase (AspAT; EC 2.6.1.1; Cormay, Łomianki, Poland; Cat No. 1-222), creatine kinase (CK; EC 2.7.3.2; Cormay, Łomianki, Poland; Cat No. 1-220), and lactate dehydrogenase (LDH; EC 1.1.1.27; Cormay, Łomianki, Poland; Cat No. 1-239) activities were measured using an Accent 220S automatic biochemical analyser (Cormay, Łomianki, Poland). The parameters were related to the number of cellular components, such as leucocyte (WBC), granulocyte (GRA), lymphocyte (LYM), monocyte (MON), erythrocyte (RBC), and platelet (PLT) counts; haemoglobin (HGB), urea, testosterone, and cortisol concentrations; and CK, LDH, AlAT, and AspAT activities (calculated by taking into account the haematocrit conversion factor). The calculation of the factor was based on the hypothetical haematocrit value of 0.500 L·L^−1^ (50%). This approach was applied to avoid misinterpretation of post-exercise blood markers results due to exercise-induced haematocrit shifts. The values of these parameters were determined according to the formula [42]:

$$calculated\;parameter\;value=\frac{measured\;parameter\;value\times 0.500}{measured\;haematocrit\;value}$$

### 2.5. Statistical Analysis

The results are presented as the mean ± standard deviation. Either repeated-measures one-way analysis of variance (ANOVA) or the Wilcoxon test (for normally and non-normally distributed data, respectively) was used to compare the CD versus KD (while consuming a CD and after consuming a KD) differences for the ICT, FGB results, and blood markers. The sample size met the research assumptions, as previously described [8]. All the blood data referring to males and data referring to individual FGB series components in males were analysed using the nonparametric Wilcoxon test. This approach is justified due to the lack of certain blood samples and data regarding particular components of detailed repetitions in individual FGB rounds of several male participants and due to the small number of participants enrolled in the analyses (5 or 6 male participants, depending on the parameter). Statistical significance was set at *p* < 0.05. Data were analysed using STATISTICA 13.3 (StatSoft Inc., Tulsa, OK, USA) software. 

## 3. Results

### 3.1. Aerobic Capacity in the ICT

**Figure 2:**
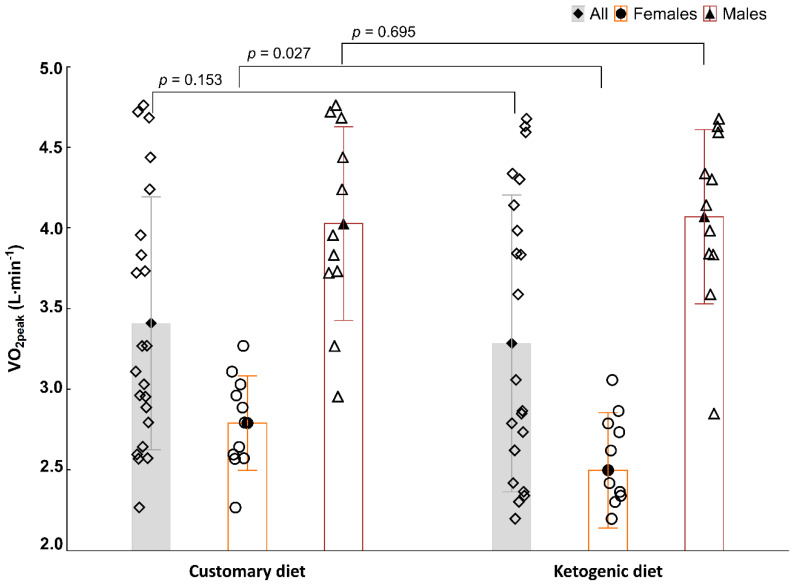
Peak oxygen uptake (VO_2peak_, L·min^−1^) in the incremental cycling test (ICT). Values are expressed as the mean (black fill) ± standard deviation and as raw data (white fill). All: ◊, females: O, males: ∆. Data were analysed by repeated-measures one-way analysis of variance. Statistical significance was set at *p* < 0.05.

There were no differences in VO_2peak_ (Figure 2), T_exh_, or W_max_ (Table 2) between consuming a CD and KD in the entire group or in males (Table 2 and Figure 2). However, in females, VO_2peak_ decreased by 10.4% and was significantly lower after consuming a KD compared with a CD (Figure 2; 2.79 ± 0.29 vs. 2.50 ± 0.36 L·min^−1^, respectively; *p* = 0.027). Nevertheless, HR_max_ was substantially higher after consuming a KD in all participants and in females (Table 2); there was no difference in males. There were no substantial differences in T_GET_, VO_2GET_, or W_GET_ between a CD and after a KD (Table 2). Moreover, HR_GET_ was higher after consuming a KD in all participants and in males, but not in females (Table 2). 

### 3.2. Discipline-Specific CrossFit Performance in the FGB Test

**Figure 3:**
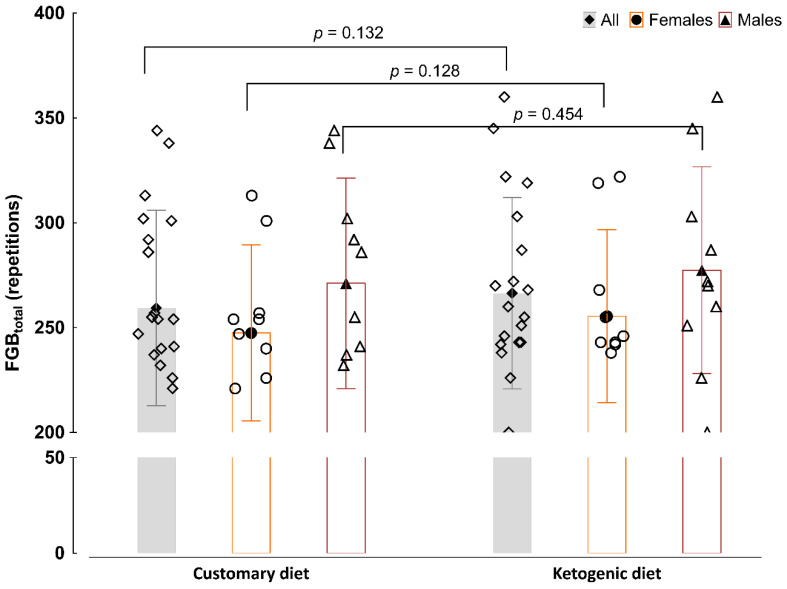
Total score in the Fight Gone Bad (FGB) test. Values are expressed as the mean (black fill) ± standard deviation and raw data (white fill). All: ◊, females: O, males: ∆. Data were analysed by repeated-measures one-way analysis of variance. Statistical significance was set at *p* < 0.05.

There were no significant differences between consuming a CD or a KD in the total score of FGB in each round (1, 2, or 3; Table S2) or the in total score when the three rounds were analysed together (Figure 3; all: *p* = 0.132; females: *p* = 0.128; males: *p* = 0.454). However, the number of repetitions in box jumps was higher in the entire group after consuming a KD in round 2, and in females after consuming a KD in rounds 2 and 3, as in the sum of box jumps in all rounds (Table S2).

### 3.3. Blood Marker Analysis

#### 3.3.1. Blood Acid–Base Balance

Regarding the entire group, the post-ICT exercise lactate concentration was significantly lower after consuming a KD compared with a CD (*p* = 0.042; Table 3). Furthermore, consuming a KD significantly affected post-ICT exercise blood acid–base balance in males. Indices such as pH (*p* = 0.028), BE (*p* = 0.028), and HCO_3_^−^ (*p* = 0.028) were significantly increased, while H^+^ (*p* = 0.028), the anion gap (*p* = 0.028), and lactate (*p* = 0.028) were substantially lower after consuming a KD (Table 3). There were no vital changes in acid–base balance parameters in females.

#### 3.3.2. Blood Biochemical Parameters

The pre-ICT exercise bilirubin concentration was elevated in the entire group after consuming a KD compared with a CD (*p* = 0.042; Table 4). Furthermore, in males, the AlAT activities measured pre-ICT (*p* = 0.043) and post-ICT (*p* = 0.043) exercise were significantly increased after consuming a KD (Table 4). Moreover, the post-ICT exercise GLU (*p* = 0.043) and BIL (*p* = 0.043) concentrations were higher after consuming a KD compared with a CD. There were no substantial changes in the other biochemical parameters in males. None of the studied markers differed after consuming a KD or a CD when considering only females or the entire group. 

#### 3.3.3. Blood Haematological Markers

Regarding the blood cell counts, after consuming a KD, there was a significant increase in the pre-exercise MON count in the entire group (*p* = 0.005; Table 5) and in females (*p* = 0.036), and in the post-exercise MON count in the entire group (*p* = 0.017) and in males (*p* = 0.043). Pre- and post-ICT exercise HGB concentrations and mean corpuscular haemoglobin (MCH) or mean corpuscular haemoglobin concentration (MCHC) were decreased after consuming a KD in the entire group (pre-ICT: HGB, *p* = 0.002; MCH, *p* = 0.001; MCHC, *p* = 0.002/post-ICT: HGB, *p* = 0.004; MCH, *p* = 0.003; MCHC, *p* = 0.004) and in females (pre-ICT: HGB, *p* = 0.003; MCH, *p* = 0.012; MCHC, *p* = 0.004/post-ICT: HGB, *p* = 0.013; MCH, *p* = 0.004; MCHC, *p* = 0.005). After consuming a KD, there was a substantial increase in the pre- and post-ICT exercise plateletcrit (PCT) in the entire group (*p* = 0.035; *p* = 0.019). The pre-exercise PDW in males was substantially lower after consuming a KD compared with a CD (*p* = 0.043).

## 4. Discussion

In the present study, we investigated the influence of a 4-week KD on aerobic capacity, select blood markers, and CrossFit-specific performance in female and male CrossFit practitioners. We found that consuming a KD had a rather slight impact on aerobic capacity as determined by monitoring cardiorespiratory indices during the ICT, and had no relevant effect on CrossFit-specific performance based on the FGB test. Consuming a KD affected blood parameters related to bilirubin and haemoglobin concentrations, MCH, and MCHC, and resulted in alterations in AlAT activity and blood acid–base balance but had a relatively minor effect on other biochemical blood indices. However, there were recognisable sex differences regarding changes in blood markers after consuming a KD. 

The data on the influence of consuming a KD on aerobic capacity in CrossFit practitioners are lacking. Moreover, studies on other endurance sport disciplines have provided conflicting results. In our current study, we found that consuming a KD significantly decreased VO_2peak_ in females. There were no changes observed in males. Zając et al. [43] revealed a significant increase in relative values of VO_2max_ and VO_2_ at lactate threshold (LT) after a 4-week KD in off-road cyclists. Burke et al. [32] reported that 3 weeks of a KD combined with intensified training and mild energy deficit, similarly to diets that either had continuously or periodically high carbohydrate availability, resulted in an increase in VO_2peak_ in elite walkers. Moreover, there was an increase in fat oxidation capacity in the KD group, but no improvement in endurance performance [32]. A pilot case study in endurance athletes by Zinn et al. [44] showed that after 10 weeks of a KD, all participants exhibited a decrease in T_exh_ in the ICT. Moreover, there was a decrease in VO_2max_ in four out of five participants, two participants experienced a decrease in the ventilatory threshold (another two maintained the baseline score), and four subjects noted a decrease in peak power [44]. It seems that the aforementioned changes may be explained by the metabolic alterations and, in particular, the reduction in carbohydrate oxidation. Shaw et al. [17] demonstrated that 31 days of consuming a KD preserved the mean submaximal exercise capacity without requiring carbohydrate restoration or supplementation (no changes in VO_2max_ or T_exh_), but they noted impaired exercise efficiency at intensities above 70% VO_2max_. Furthermore, no effect of consuming a KD on various indicators of aerobic capacity (e.g., VO_2max_, VO_2peak_) has been noted in few other studies [45,46,47]. Increases in VO_2max_ may be partially explained by decreases in body weight and improvements in participant’s aerobic capacity. In turn, decreases in VO_2max_ are likely due to changes in metabolic pathways that impair glycogen metabolism at higher exercise intensities. Furthermore, we also registered that consuming a KD greatly impacted HR during the ICT. HR_max_ was increased in the entire group and in females, while HR_GET_ was increased in the entire group and in males. However, HR alterations were not related to the T_exh_, W_max_, T_GET_, and W_GET_ improvement during the ICT. Thus, it is difficult to speculate that the CrossFitters’ adaptation level in terms of the ICT aerobic capacity and anaerobic threshold changes actually improved. In our opinion, only an increase in HR with simultaneous performance and/or workload improvement may be considered a beneficial and desired effect. 

We would like to emphasise that, to our best knowledge, only our previous [8] and two other papers [46,48] have analysed the effect of KD in CrossFit practitioners. In our study, it was revealed that consuming a KD had no relevant effect on CrossFit-specific performance. After consuming a KD, the athletes showed improvement in only one exercise in the FGB (box jumps results in second (entire group) and second and third (females) round). However, fluctuations in a single component of the FGB test cannot stand as a reflection of changes in discipline-specific performance as a whole. Gregory et al. [48] found no advantage of a 6-week KD (males, *n* = 3; females, *n* = 9) over the control customary diet (males, *n* = 2; females, *n* = 13) combined with training on power (vertical and standing long jumps) and CrossFit performance (total performance time in exercises consisting of a 500 m row, 40 body weight squats, 30 abdominal mat sit-ups, 20 hand release push-ups, and 10 pull-ups), although performance was improved after both diets. Nevertheless, the KD protocol implemented in that study was slightly different than ours (<10% of energy from carbohydrates). Kephart et al. [46] found no effect of a 12-week KD on various performance indices (one-repetition-maximum squat and power clean, maximum push-up repetitions, 400 m run time, and VO_2peak_ values assessed with a graded treadmill test) in recreationally trained CrossFit practitioners (KD group: 5 males and 2 females; control group: 4 males and 1 female). Thus, these findings are similar to our current study. It should be noted, however, that in both of the aforementioned studies, there was a small sample size in the KD diet group, especially for female CrossFitters. 

To the best of our knowledge, the current study is also the first to investigate the effect of consuming a KD on the CrossFit-specific cross-sectional FGB test and undertake an in-depth analysis of aerobic capacity indicators in the ICT with simultaneous control of diagnostically significant haematological, hormonal, muscular, and metabolic function markers and acid–base balance blood indices before and after progressive exercise. In addition, the possibility of verifying the impact of this diet depending on the participant’s sex is particularly valuable.

Even small changes to acid–base balance may place substantial stress on the body’s buffering mechanisms [49]. Previous studies [50,51] have indicated that there is a tendency towards lower blood pH and reduced HCO_3_^−^ concentrations and blood base excess after a KD at rest and after exercise. The results of our study are in contrast to the previous findings. We found no differences between the diets in blood pH, H^+^, and HCO_3_^−^ concentration or BE when analysing the entire group. When analysing males only, there were even favourable changes after consuming a KD compared with a CD in post-exercise parameters of blood acid–base balance (elevated values of pH, BE, and HCO_3_^−^, along with a lower H^+^ concentration). Still, the results referring to males need to be interpreted carefully because of the small number of analysed samples (*n* = 5). Nevertheless, the results of our study are comparable to the findings of Carr et al. [49]. They found no differences in blood pH between ketogenic low-carbohydrate, high-fat or periodised carbohydrate groups at any time point, and no significant differences in pre-exercise or post-exercise (graded maximal exercise treadmill test) HCO_3_^−^ concentrations between groups. It is important to note that consuming a KD may have no influence on resting or exercise-associated blood indices of acid–base status. This is probably due to the long duration of KD consumption, which was sufficient for acids to be neutralised by buffering systems in blood, respiratory, and renal systems or because of the participants’ training adaptation [49]. In our study, we found significant decrease in the post-exercise lactate concentration after consuming a KD in the entire group and in males. Only one previous study by Zając et al. [43] also showed a lower plasma lactate concentration at rest, during and after an exercise protocol (varied intensity: 90 min at 85% of the LT and 15 min with a continuous effort at 115% of the LT on a cycloergometer) after consuming a KD. Other studies investigating lactate have demonstrated higher concentrations during exercise [52,53] or no effect on lactate at rest [47,52,53,54] and during exercise [17,32,54,55] after consuming a KD compared with a control diet. It is not fully understood if the lactate concentration changes depending on an inhibited lactate efflux from working muscles due to reduced blood buffering capacity or a decreased rate of glycolysis [56]. 

Furthermore, in our study, we found substantial increase in the post-exercise GLU concentration in males after consuming a KD. However, as mentioned earlier, blood markers in males needs to be interpreted cautiously due to the small sample size. Other studies have shown conflicting results in this measure. Burke et al. [32], Phinnney et al. [47] and Urbain et al. [57] revealed a lower GLU concentration at rest. Helge et al. [52] showed a higher GLU concentration at exhaustion after a low-carbohydrate, fat-rich diet. However, Burke et al. [32] reported a lower GLU concentration during exercise. Other studies on physically active people have shown no differences between groups or no effect of a KD on GLU concentration at rest [43,45,46,47,52,53,54,58] or during exercise [17,43,53,54,55].

Few studies have measured concentrations of hormones or enzyme activity in relation to consuming a KD. One study found no significant effect of consuming a KD on testosterone and cortisol concentrations [43], while another study noted that consuming a KD enhanced pre- and post-exercise concentrations of cortisol [50]. There was also no effect of consuming a KD on CK or LDH activities at rest and during exercise [43,59]. Similarly, there were no relevant differences in the abovementioned indices in our study. Still, significant increase in pre- and post-ICT exercise AlAT activity in males were noted. It is important to note that aminotransferase activities may increase after exercise because they are released from activated muscles (AspAT mainly from muscles and AlAT mainly from the liver). Their activities may increase with the increasing intensity and duration of exercise [60]. However, in our opinion, AlAT activity fluctuations (without simultaneous AspAT and CK alterations) suggest that consuming a KD may be somehow related to liver overload in males. This idea might also be supported through the elevated bilirubin concentration that was registered after participants consumed a KD. Human and animal studies have demonstrated that a higher bilirubin concentration has protective potential in some conditions (e.g., nonalcoholic fatty liver disease) [61,62]. In addition, bilirubin changes after consuming a KD may partially explain the lower HGB concentration in the blood (in the entire group and in females) via expanded haemolytic activity (taking into account that total and indirect bilirubin are often higher in athletic populations) [60]. Moreover, slight differences in hormone concentrations due to the dietary intervention may be explained by mechanisms controlling the hormonal and metabolic responses to exercise. The depletion of glycogen leads to the stimulation of glucose production and lipolysis based on the changes in the secretion of glucoregulatory hormones [63]. However, these aforementioned hypotheses are intended to consider new directions in the KD field and should be confirmed through further research.

Although there were certain statistically significant changes in haematological blood markers, not all of them seem to be clinically relevant. However, a substantial decrease in selected red cell parameters (HGB, MCH, and MCHC) was found at rest and post-exercise in the entire group and in females. The results are quite surprising because a KD would likely include foods characterised by high content of iron that can be well absorbed (e.g., red meat). Moreover, the mean dietary iron intake was slightly higher after consuming a KD compared with a CD in both females (~13.4 vs. ~14.1 mg·day^−1^; Table S1) and males (~13.8 vs. ~15.8 mg·day^−1^, Table S1). Still, in females, the daily iron intake from consuming a CD or a KD was lower than the dietary recommendations for athletes [4] and for the Polish population [64]. When not taking into account the haematocrit conversion factor, the pre-exercise HGB concentrations in females were ~8.41 mmol·L^−1^ after consuming a CD and ~8.39 mmol·L^−1^ after consuming a KD and fell within the reference range (7.4–9.9 mmol·L^−1^ [65]). Another issue is the fluctuations in haematological status measures during the menstrual cycle [66], which we did not take into account in the current study. Nevertheless, the protocol lasted 4 weeks, which should mean that the measurements were taken in the comparable individual menstrual cycle phases. A similar situation was described in another study by Burke et al. [28]. Furthermore, the latest systematic review and meta-analysis indicates that general guidelines on exercise performance across the menstrual cycle cannot be formed (with the exception that exercise test performance may be slightly impaired during the early follicular phase of the menstrual cycle compared with all other phases) [67]. However, in this regard, the individual response seems to be finally crucial. The decrements in select haematological parameters (e.g., depicting the so-called ‘iron status’) in females were slightly reflected in aerobic capacity, namely the decrease in VO_2peak_ after consuming a KD_._ Moreover, we noted an increase in MON and PCT counts after the KD. Still, the parameters were within reference levels after consuming either studied diet. 

It should be noted that the length of KD treatment determines whether any adaptive response can be achieved. Although an increase in KBs is usually observed (with constant gluconeogenesis) after 2–3 days of KD intake, the carbohydrate energy sources are vitally depleted, which leads to deterioration of training capacity/well-being and performance in moderate-intensity (<70% VO_2peak_) and high-intensity (>80% VO_2peak_) efforts [29]. However, with prolonged KD consumption, certain metabolic adaptations are observed: in the short term (5–10 days), decreased glycogen and active form of pyruvate dehydrogenase and increased intramuscular triglycerides, hormone sensitive lipase, fatty acid translocase, and carnitine palmitoyltransferase; in the medium term (3–6 weeks), consolidation of the short-term metabolic adaptation; and/or in the long term (>3–4 months), it is unclear whether additional changes are possible than those already obtained during the medium-term period. Although training capacity/well-being and performance in moderate training intensity (<70% VO_2peak_) stabilise after an initial decrease, their continued deterioration may be observed at higher intensities [29].

The unquestionable strength of our study is the novel exploration of how sex influences the effects of consuming a KD: we observed that males and females responded differently to consuming a KD. It has been demonstrated that there may be some sex bias in cohorts included in sport medicine, nutrition, and supplementation research [8,68,69]. Based on our results, it is speculated that there may be certain factors limiting the applicable effectiveness of a KD in females. Moreover, the 4-week KD period was sufficient to obtain the medium-term adaptation necessary for actual assessment of the potential benefits of consuming a KD in functional training [8,29]. Additional strengths of the study are that only regularly trained CrossFitters (≥4 CrossFit training units per week, ≥2 years of CrossFit training experience) participated in this study. Moreover, the applied research methods provide highly accurate and reliable data (e.g., aerobic capacity and discipline-specific performance analyses, monitoring of various blood biomarkers, and diet compliance control). Finally, obtained results may also be extrapolated into other sports disciplines, especially where high-intensity functional training programs are implemented.

Our study has some limitations, which we have thoroughly described and discussed in our previous paper [8]. Briefly, a main limitation is the lack of menstrual cycle phase monitoring and the lack of a control group. Nevertheless, general guidelines on exercise performance across the menstrual cycle cannot be formed [67]. When considering the issue of the lack of a control group, it should be remembered that (a) blinding was impossible because it is difficult to blind a KD; (b) we were interested mainly in the changes before (during CD) and after the implementation of KD model; and (c) the application of a high-carbohydrate diet has been extensively investigated, and it is clear that this nutritional model is efficient and can support exercise performance during functional training [3,6,70,71]. Moreover, the individual variation in vulnerability to consuming a KD and the willingness to continue consuming a KD should be taken into consideration when prescribing a KD. The limitation of the current study is also a low number of male participants (*n* = 5 or 6 depending on the parameter) included in the statistical analysis of blood parameters and results within each FGB round (for those participants, the repetitions from all stations and all rounds were recorded together). To avoid misleading conclusions, we tested all blood parameter data in males using nonparametric statistical tests. Moreover, the results of these analyses were interpreted with caution. Finally, we hypothesise that the lack of an increase in aerobic capacity during a 4-week training cycle, or even an observed tendency to decrease especially in females, discredits, to a large extent, this nutritional model as a means to effectively support high-intensity functional training. However, it refers to training targeted toward increasing the potential of physical adaptation and exercise performance.

## 5. Conclusions

Consuming a KD has no advantage in CrossFit training with respect to aerobic capacity and discipline-specific performance. Compared with a baseline customary diet, a 4-week KD slightly impaired aerobic capacity in females, but not in males. Regardless of sex, consuming a KD had no vital effect on discipline-specific performance (despite certain box jump improvement). In addition, the pre-exercise bilirubin concentration was elevated in the entire group after consuming a KD. A KD may also contribute to increased risk of certain haematological disturbances in female CrossFit athletes. In males, there was an undesirable AlAT elevation with a small tendency towards improved acid–base balance. In summary, we cannot recommend consuming a KD as an effective nutritional strategy in functional training aimed at improving athletic performance. Furthermore, when adhering to a KD, haematological markers in female athletes and liver function status in male athletes should be monitored. However, our study also demonstrated that training while consuming a KD is practically possible and is not associated with the occurrence of any severe disruption in homeostasis as evaluated on the basis of blood testing or the lack of spectacular aerobic adaptation or exercise performance decrease.

## Figures and Tables

**Table 1:** Anthropometric characteristics.

Indicator	Diet Type	All	Females	Males
*n*	-	22	11	11
Age (years)	-	29.5 ± 4.4	30.5 ± 3.3	28.5 ± 5.3
Body height (cm)	-	172 ± 8	166 ± 7	177 ± 5
Body mass (kg)	Customary diet Ketogenic diet	71.1 ± 12.8 70.1 ± 12.6	60.8 ± 4.9 60.0 ± 4.4	81.3 ± 9.5 80.1 ± 9.7

Note: Values are expressed as the mean ± standard deviation.

**Table 2:** Incremental cycling test (ICT) results.

Indicator	Diet Type	All	Females	Males
T_exh_ (s)	Customary diet Ketogenic diet *p*	636 ± 178 634 ± 182 0.903 *	510 ± 104 496 ± 102 0.485 *	761 ± 146 771 ± 134 0.706 *
W_max_ (W)	Customary diet Ketogenic diet *p*	252 ± 61 248 ± 60 0.388 **	205 ± 29 198 ± 26 0.345 **	300 ± 43 298 ± 38 0.753 **
HR_max_ (bpm)	Customary diet Ketogenic diet *p*	181 ± 9 184 ± 8 **0.042** *	176 ± 6 180 ± 6 **0.014** *	185 ± 10 187 ± 8 0.447 *
T_GET_ (s)	Customary diet Ketogenic diet *p*	486 ± 122 479 ± 126 0.414 *	401 ± 79 386 ± 77 0.082 *	571 ± 95 572 ± 92 0.936 *
VO_2GET_ (L·min^−1^)	Customary diet Ketogenic diet *p*	2.68 ± 0.63 2.64 ± 0.72 0.524 *	2.18 ± 0.34 2.03 ± 0.26 0.105 *	3.19 ± 0.41 3.26 ± 0.43 0.388 *
%VO_2maxGET_	Customary diet Ketogenic diet *p*	82.7 ± 6.4 83.8 ± 8.5 0.592 **	83.9 ± 5.7 83.2 ± 9.7 0.929 **	81.4 ± 7.1 84.5 ± 7.6 0.385 *
W_GET_ (W)	Customary diet Ketogenic diet *p*	210 ± 44 211 ± 49 0.747 *	175 ± 19 170 ± 27 0.463 **	245 ± 31 252 ± 26 0.082 *
HR_GET_ (bpm)	Customary diet Ketogenic diet *p*	171 ± 7 173 ± 7 **0.044** *	170 ± 7 171 ± 4 0.615 *	171 ± 8 175 ± 8 **0.029** *

Note: Values are expressed as the mean ± standard deviation. * Data were analysed by repeated-measures one-way analysis of variance. ** Data were analysed by the Wilcoxon signed-rank test. Statistical significance was set at *p* < 0.05. Abbreviations: HR_GET_, heart rate at gas exchange threshold; HR_max_, maximum value of heart rate during incremental cycling test; T_exh_, time to exhaustion; T_GET_, time to reach gas exchange threshold; VO_2GET_, oxygen uptake at gas exchange threshold; W_GET_, workload at gas exchange threshold; W_max_, maximum ICT workload; %VO_2maxGET_, percentage of maximum oxygen uptake at gas exchange threshold.

**Table 3:** Blood acid–base balance.

Indicator	Diet Type	Pre-ICT Exercise			Post-ICT Exercise		
		All	Females	Males	All	Females	Males
pH	Customary diet Ketogenic diet *p*	7.405 ± 0.027 7.404 ± 0.023 0.895 *	7.407 ± 0.021 7.405 ± 0.017 0.732 *	7.402 ± 0.038 7.404 ± 0.033 0.753 **	7.239 ± 0.056 7.257 ± 0.046 0.178 *	7.253 ± 0.059 7.259 ± 0.038 0.676 *	7.213 ± 0.044 7.253 ± 0.062 **0.028** **
H^+^ (mmol·L^−1^)	Customary diet Ketogenic diet *p*	39.41 ± 2.49 39.45 ± 2.02 0.944 *	39.21 ± 1.95 39.42 ± 1.51 0.764 *	39.77 ± 3.46 39.52 ± 2.92 0.753 **	58.16 ± 7.55 55.61 ± 6.00 0.155 *	56.30 ± 7.82 55.24 ± 4.96 0.569 *	61.57 ± 6.22 56.28 ± 8.07 **0.028** **
BE (mmol·L^−1^)	Customary diet Ketogenic diet *p*	−0.3 ± 1.8 −0.7 ± 2.0 0.502 *	−0.3 ± 1.9 −1.0 ± 2.1 0.411 *	−0.2 ± 1.8 −0.1 ± 1.6 0.753 **	−13.8 ± 2.7 −13.1 ± 2.4 0.351 *	−13.5 ± 2.7 −13.1 ± 2.2 0.569 *	−14.4 ± 3.0 −13.1 ± 2.9 **0.028** **
HCO_3_^−^ (mmol·L^−1^)	Customary diet Ketogenic diet *p*	24.2 ± 1.4 23.9 ± 1.4 0.524 *	24.2 ± 1.4 23.7 ± 1.5 0.393 *	24.2 ± 1.4 24.4 ± 1.2 0.753 **	15.0 ± 1.7 15.4 ± 1.5 0.384 *	15.2 ± 1.7 15.4 ± 1.4 0.620 *	14.8 ± 1.7 15.5 ± 1.8 **0.028** **
Anion gap (mmol·L^−1^)	Customary diet Ketogenic diet *p*	10.6 ± 1.6 10.8 ± 1.8 0.501 **	10.9 ± 1.8 10.8 ± 2.0 0.878 **	10.1 ± 1.0 10.9 ± 1.4 0.208 **	20.7 ± 5.9 22.0 ± 2.2 0.737 **	19.8 ± 7.2 21.8 ± 2.2 0.610 **	22.5 ± 1.7 22.4 ± 2.4 **0.028** **
Lactate (mmol·L^−1^)	Customary diet Ketogenic diet *p*	1.8 ± 0.5 1.8 ± 0.3 0.568 *	1.7 ± 0.3 1.8 ± 0.2 0.758 *	2.1 ± 0.7 1.8 ± 0.3 0.345 **	12.4 ± 2.6 11.1 ± 2.1 **0.042** *	11.6 ± 2.7 10.8 ± 1.9 0.215 *	13.9 ± 1.6 11.7 ± 2.4 **0.028** **
Osmolarity (mmol·kg^−1^)	Customary diet Ketogenic diet *p*	290.7 ± 2.8 289.7 ± 3.9 0.362 *	290.9 ± 3.1 289.9 ± 3.7 0.299 *	290.2 ± 2.4 289.4 ± 4.5 0.917 **	293.0 ± 2.5 292.7 ± 4.4 0.819 *	292.8 ± 2.8 292.7 ± 4.7 0.953 *	293.5 ± 2.0 292.8 ± 4.1 0.116 **

Note: Values are expressed as the mean ± standard deviation. * Data were analysed by repeated-measures one-way analysis of variance. ** Data were analysed by the Wilcoxon signed-rank test. Statistical significance set at *p* < 0.05. Abbreviations: BE, standard base excess; H^+^, hydrogen ion concentration; HCO_3_^−^, bicarbonate ion concentration; ICT, incremental cycling test.

**Table 4:** Select hormonal, muscular, and metabolic function markers.

Indicator	Diet Type	Pre-ICT Exercise			Post-ICT Exercise		
		All	Females	Males	All	Females	Males
Testosterone (ng·mL^−1^)	Customary diet Ketogenic diet *p*	2.64 ± 2.15 3.02 ± 2.43 0.569 **	1.31 ± 0.37 1.74 ± 1.66 0.594 **	5.59 ± 1.09 5.84 ± 0.88 0.500 **	2.72 ± 2.27 3.16 ± 2.63 0.982 **	1.27 ± 0.37 1.75 ± 1.79 0.534 **	5.90 ± 0.75 6.25 ± 0.67 0.500 **
Cortisol (ng·mL^−1^)	Customary diet Ketogenic diet *p*	306.7 ± 108.9 293.7 ± 97.5 0.756 **	314.1 ± 127.7 297.3 ± 106.5 0.639 *	290.4 ± 56.8 285.7 ± 84.7 0.893 **	362.1 ± 126.3 326.5 ± 109.8 0.183 *	359.4 ± 133.4 337.0 ± 123.0 0.537 *	368.1 ± 123.7 303.3 ± 80.2 0.080 **
T/C ratio	Customary diet Ketogenic diet *p*	0.0096 ± 0.0077 0.0084 ± 0.0081 0.079 **	0.0049 ± 0.0026 0.0039 ± 0.0015 0.101 *	0.0197 ± 0.0045 0.0181 ± 0.0083 0.500 **	0.0111 ± 0.0099 0.0108 ± 0.0102 0.501 **	0.0059 ± 0.0040 0.0054 ± 0.0044 0.534 **	0.0225 ± 0.0094 0.0226 ± 0.0094 0.893 **
Creatine kinase (U·L^−1^)	Customary diet Ketogenic diet *p*	318.0 ± 242.6 250.2 ± 223.5 0.469 **	280.7 ± 260.4 144.2 ± 76.0 0.110 **	400.2 ± 197.3 483.3 ± 272.1 0.345 **	362.6 ± 310.8 277.3 ± 245.3 0.717 **	313.8 ± 334.6 163.5 ± 84.1 0.248 **	469.9 ± 247.8 527.7 ± 306.6 0.345 **
LDH (U·L^−1^)	Customary diet Ketogenic diet *p*	425 ± 78 402 ± 55 0.293 *	443 ± 66 402 ± 55 0.075 **	386 ± 95 403 ± 63 0.893 **	470 ± 95 448 ± 68 0.416 *	478 ± 79 454 ± 64 0.594 **	454 ± 133 436 ± 83 0.686 **
Glucose (mg·dL^−1^)	Customary diet Ketogenic diet *p*	89 ± 9 87 ± 9 0.592 *	86 ± 7 85 ± 9 0.699 *	93 ± 11 91 ± 9 0.753 **	118 ± 19 121 ± 20 0.541 *	121 ± 21 123 ± 17 0.728 *	111 ± 13 116 ± 25 **0.043** **
Urea (mmol·L^−1^)	Customary diet Ketogenic diet *p*	6.5 ± 1.5 7.3 ± 2.1 0.108 *	7.0 ± 1.5 7.4 ± 2.4 0.482 *	5.5 ± 0.8 7.3 ± 1.8 0.080 **	6.4 ± 1.2 7.1 ± 1.9 0.118 *	6.7 ± 1.3 7.2 ± 2.3 0.452 *	5.8 ± 0.9 7.1 ± 1.2 0.080 **
AlAT (U·L^−1^)	Customary diet Ketogenic diet *p*	18.0 ± 4.3 19.8 ± 6.9 0.279 *	17.9 ± 4.5 17.1 ± 4.5 0.663 *	18.4 ± 4.4 25.8 ± 7.9 **0.043** **	23.1 ± 6.5 24.7 ± 5.9 0.134 **	22.4 ± 7.1 22.4 ± 4.8 0.534 **	24.7 ± 5.3 29.9 ± 5.2 **0.043** **
AspAT (U·L^−1^)	Customary diet Ketogenic diet *p*	35.3 ± 11.1 33.2 ± 7.4 0.569 **	34.9 ± 13.0 30.4 ± 5.6 0.374 **	36.1 ± 6.6 39.2 ± 7.7 0.893 **	38.4 ± 13.9 36.1 ± 7.3 0.642 **	37.7 ± 16.8 33.3 ± 6.2 1.000 **	39.9 ± 3.0 42.1 ± 6.4 0.345 **
Bilirubin (μmol·L^−1^)	Customary diet Ketogenic diet *p*	10 ± 5 14 ± 9 **0.042** *	12 ± 4 15 ± 9 0.248 *	6 ± 4 11 ± 8 0.059 **	19 ± 8 20 ± 8 0.709 *	20 ± 9 19 ± 9 0.786 *	20 ± 9 19 ± 9 **0.043** **

Note: Values are expressed as the mean ± standard deviation. * Data were analysed by repeated-measures one-way analysis of variance. ** Data were analysed by the Wilcoxon signed-rank test. Statistical significance was set at *p* < 0.05. Abbreviations: AlAT, alanine aminotransferase; AspAT, aspartate aminotransferase; ICT, incremental cycling test; LDH, lactate dehydrogenase; T/C ratio, testosterone-to-cortisol ratio.

**Table 5:** Blood haematological parameters.

Indicator	Diet Type	Pre-ICT Exercise			Post-ICT Exercise		
		All	Females	Males	All	Females	Males
WBC (10^9^·L^−1^)	Customary diet Ketogenic diet *p*	7.2 ± 1.7 7.7 ± 2.1 0.359 *	7.5 ± 1.8 8.1 ± 2.3 0.409 *	6.5 ± 1.5 6.7 ± 1.3 0.686 **	11.5 ± 2.3 11.9 ± 3.4 0.540 *	12.2 ± 2.1 12.8 ± 3.7 0.556 *	9.9 ± 2.3 10.0 ± 1.2 0.893 **
LYM (10^9^·L^−1^)	Customary diet Ketogenic diet *p*	2.7 ± 0.7 2.7 ± 0.5 0.920 *	2.8 ± 0.7 2.7 ± 0.6 0.821 *	2.5 ± 0.7 2.6 ± 0.3 0.893 **	5.2 ± 1.3 5.1 ± 1.0 0.889 *	5.4 ± 1.2 5.3 ± 1.1 0.878 **	4.6 ± 1.4 4.7 ± 0.8 0.893 **
MON (10^9^·L^−1^)	Customary diet Ketogenic diet *p*	0.5 ± 0.2 0.6 ± 0.2 **0.005** **	0.5 ± 0.2 0.6 ± 0.2 **0.036** *	0.4 ± 0.1 0.5 ± 0.1 0.080 **	0.7 ± 0.2 0.9 ± 0.3 **0.017** *	0.8 ± 0.2 1.0 ± 0.3 0.056 *	0.6 ± 0.2 0.7 ± 0.1 **0.043** **
GRA (10^9^·L^−1^)	Customary diet Ketogenic diet *p*	4.1 ± 1.2 4.4 ± 1.7 0.423 *	4.3 ± 1.2 4.8 ± 1.9 0.412 *	3.6 ± 1.3 3.6 ± 1.2 0.893 **	5.6 ± 1.4 5.9 ± 2.7 0.733 **	6.0 ± 1.2 6.5 ± 2.9 0.575 **	4.7 ± 1.7 4.6 ± 1.3 0.893 **
LYM (%)	Customary diet Ketogenic diet *p*	37.7 ± 8.1 36.4 ± 8.7 0.537 *	36.8 ± 5.7 35.1 ± 8.7 0.500 *	39.5 ± 12.5 39.3 ± 8.8 0.686 **	44.9 ± 8.4 44.3 ± 9.3 0.750 *	44.2 ± 6.4 42.9 ± 8.8 0.523 *	46.5 ± 12.6 47.3 ± 10.7 0.893 **
MON (%)	Customary diet Ketogenic diet *p*	6.2 ± 1.3 7.6 ± 1.2 **0.013** *	6.3 ± 1.5 7.5 ± 1.4 0.087 *	6.2 ± 0.5 7.7 ± 0.8 0.080 **	6.4 ± 1.0 7.5 ± 0.9 **0.019** *	6.2 ± 1.2 7.5 ± 0.9 **0.016** *	6.6 ± 0.7 7.5 ± 1.1 0.345 **
GRA (%)	Customary diet Ketogenic diet *p*	56.1 ± 8.3 56.0 ± 9.1 0.973 *	56.9 ± 6.1 57.4 ± 9.1 0.856 *	54.3 ± 12.5 53.0 ± 9.4 0.893 **	48.7 ± 8.6 48.2 ± 9.6 0.808 *	49.6 ± 6.5 49.6 ± 9.2 0.989 *	46.9 ± 12.9 45.2 ± 10.8 0.686 **
RBC (10^12^·L^−1^)	Customary diet Ketogenic diet *p*	5.87 ± 0.26 5.85 ± 0.27 0.156 *	5.84 ± 0.28 5.82 ± 0.30 0.221 *	5.92 ± 0.21 5.90 ± 0.18 0.500 **	5.79 ± 0.24 5.80 ± 0.27 0.715 *	5.76 ± 0.26 5.77 ± 0.29 0.798 *	5.86 ± 0.19 5.87 ± 0.21 0.500 **
HGB (mmol·L^−1^)	Customary diet Ketogenic diet *p*	11.15 ± 0.22 10.76 ± 0.32 **0.002** *	11.19 ± 0.19 10.71 ± 0.36 **0.003** *	11.04 ± 0.26 10.88 ± 0.21 0.345 **	11.01 ± 0.21 10.68 ± 0.35 **0.004** *	11.01 ± 0.22 10.58 ± 0.34 **0.013** **	11.03 ± 0.22 10.89 ± 0.32 0.345 **
HCT (L·L^−1^)	Customary diet Ketogenic diet *p*	0.398 ± 0.047 0.403 ± 0.024 0.577 *	0.376 ± 0.028 0.392 ± 0.016 0.113 *	0.447 ± 0.044 0.428 ± 0.018 0.225 **	0.413 ± 0.039 0.418 ± 0.038 0.506 *	0.405 ± 0.038 0.405 ± 0.037 0.997 *	0.429 ± 0.038 0.447 ± 0.022 0.500 **
MCV (fL)	Customary diet Ketogenic diet *p*	85.4 ± 3.7 85.7 ± 3.9 0.196 *	85.8 ± 4.1 86.1 ± 4.4 0.237 *	84.6 ± 3.0 84.8 ± 2.5 0.500 **	86.2 ± 3.8 86.3 ± 3.9 0.340 *	86.5 ± 4.2 86.8 ± 4.3 0.169 *	85.4 ± 2.8 85.3 ± 3.0 0.500 **
MCH (fmol)	Customary diet Ketogenic diet *p*	1.90 ± 0.10 1.84 ± 0.09 **0.001** *	1.92 ± 0.11 1.84 ± 0.10 **0.012** *	1.87 ± 0.06 1.84 ± 0.05 0.285 **	1.90 ± 0.10 1.84 ± 0.10 **0.003** *	1.91 ± 0.11 1.84 ± 0.11 **0.004** *	1.88 ± 0.07 1.86 ± 0.08 0.418 **
MCHC (mmol·L^−1^)	Customary diet Ketogenic diet *p*	22.25 ± 0.43 21.51 ± 0.65 **0.002** *	22.33 ± 0.38 21.40 ± 0.72 **0.004** *	22.08 ± 0.54 21.76 ± 0.41 0.944 **	22.08 ± 0.43 21.36 ± 0.71 **0.004** *	22.09 ± 0.45 21.17 ± 0.68 **0.005** *	22.06 ± 0.42 21.77 ± 0.63 0.345 **
RDW (%)	Customary diet Ketogenic diet *p*	12.9 ± 1.0 13.0 ± 0.8 0.386 *	12.7 ± 1.1 12.8 ± 0.8 0.407 *	13.2 ± 0.5 13.3 ± 0.9 0.787 **	13.0 ± 1.0 13.2 ± 0.9 0.196 *	12.9 ± 1.1 13.1 ± 1.0 0.294 *	13.1 ± 0.8 13.3 ± 0.5 0.418 **
PLT (10^9^·L^−1^)	Customary diet Ketogenic diet *p*	248 ± 76 266 ± 81 0.289 *	259 ± 86 277 ± 95 0.451 *	225 ± 47 243 ± 31 0.345 **	265 ± 77 305 ± 95 0.070 *	270 ± 90 324 ± 100 0.059 *	253 ± 43 264 ± 77 0.893 **
MPV (fL)	Customary diet Ketogenic diet *p*	8.7 ± 0.8 8.9 ± 0.9 0.157 *	8.7 ± 0.9 8.9 ± 1.0 0.212 *	8.7 ± 0.6 8.9 ± 0.9 0.787 **	8.9 ± 0.8 9.1 ± 0.9 0.179 *	8.9 ± 0.9 9.1 ± 1.0 0.378 *	8.7 ± 0.8 9.0 ± 0.9 0.273 **
PCT (cL·L^−1^)	Customary diet Ketogenic diet *p*	0.166 ± 0.035 0.186 ± 0.040 **0.035** *	0.163 ± 0.041 0.186 ± 0.044 0.085 *	0.172 ± 0.020 0.186 ± 0.032 0.225 **	0.189 ± 0.047 0.225 ± 0.053 **0.019** *	0.190 ± 0.056 0.232 ± 0.054 0.059 *	0.186 ± 0.021 0.210 ± 0.053 0.138 **
PDW (%)	Customary diet Ketogenic diet *p*	12.6 ± 1.2 12.8 ± 1.3 0.561 *	12.3 ± 1.3 13.0 ± 1.4 0.216 *	13.3 ± 0.8 12.5 ± 0.9 **0.043** **	12.8 ± 1.2 12.8 ± 1.1 0.845 *	12.7 ± 1.2 12.7 ± 1.2 0.967 *	13.1 ± 1.5 12.8 ± 1.2 0.787 **

Note: Values are expressed as the mean ± standard deviation (SD). * Data were analysed by repeated-measures one-way analysis of variance. ** Data were analysed by the Wilcoxon signed-rank test. Statistical significance was set at *p* < 0.05. Abbreviations: GRA, granulocyte count; HCT, haematocrit value; HGB, haemoglobin concentration; ICT, incremental cycling test; LYM, lymphocyte count; MCHC, mean corpuscular haemoglobin concentration; MCH, mean corpuscular haemoglobin; MCV, mean corpuscular volume; MON, monocyte count; MPV, mean platelet volume; PCT, plateletcrit; PDW, platelet distribution width; PLT, thrombocyte count; RBC, erythrocyte count; RDW, red blood cell distribution width; WBC, leucocyte count.

## Data Availability

The datasets used and/or analysed during the current study are available from the corresponding author on reasonable request.

## Supplementary Materials

The following are available online at https://www.mdpi.com/2072-6643/13/3/864/s1, Table S1: Detailed nutritional value of customary and ketogenic diets. Table S2. The Fight Gone Bad (FGB) test results.
