# Supplementary material for: Is a Four-Week Ketogenic Diet an Effective Nutritional Strategy in CrossFit-Trained Female and Male Athletes?
Source: Nutrients. 2021 Mar 6;13(3):864. doi: 10.3390/nu13030864 (PMC8001376; doi:10.3390/nu13030864)
Supplement: Supplementary file 1 [file nutrients-13-00864-s001.zip › Supplementary Table S1.pdf]

Table S1. Detailed nutritional value of customary and ketogenic diets.

		Females	Males
Energy (kcal·day^{-1})	Customary diet	2485 ± 279	2645 ± 289
	Ketogenic diet	2659 ± 273	2955 ± 265
Protein (g·day^{-1})	Customary diet	109 ± 26	127 ± 15
	Ketogenic diet	115 ± 17	134 ± 9
Protein (g·kg$_{BM}$·day^{-1})	Customary diet	1.8 ± 0.4	1.6 ± 0.2
	Ketogenic diet	1.9 ± 0.2	1.7 ± 0.2
Protein (% $_{energy\ intake}$)	Customary diet	17.7 ± 4.5	19.4 ± 3.0
	Ketogenic diet	17.3 ± 1.7	18.4 ± 2.8
Fat (g·day^{-1})	Customary diet	101 ± 19	107 ± 29
	Ketogenic diet	230 ± 24*	245 ± 29*
Fat (g·kg$_{BM}$·day^{-1})	Customary diet	1.7 ± 0.4	1.3 ± 0.3
	Ketogenic diet	3.8 ± 0.5*	3.2 ± 0.6*
Fat (% $_{energy\ intake}$)	Customary diet	36.9 ± 7.1	36.4 ± 8.1
	Ketogenic diet	77.8 ± 1.7*	77.0 ± 2.4*
Carbohydrate (g·day^{-1})	Customary diet	285 ± 79	294 ± 80
	Ketogenic diet	33 ± 3*	33 ± 5*
Carbohydrate (g·kg$_{BM}$·day^{-1})	Customary diet	4.7 ± 1.5	3.6 ± 0.9
	Ketogenic diet	0.5 ± 0.1*	0.4 ± 0.1*
Carbohydrate (% $_{energy\ intake}$)	Customary diet	45.4 ± 10.5	44.2 ± 9.5
	Ketogenic diet	4.9 ± 0.4*	4.5 ± 0.5*
Dietary fiber (g·day^{-1})	Customary diet	32.8 ± 11.0	34.7 ± 6.9
	Ketogenic diet	9.9 ± 2.2*	8.9 ± 1.6*
Cholesterol (mg·day^{-1})	Customary diet	491 ± 292	497 ± 318
	Ketogenic diet	670 ± 509	637 ± 282
Sucrose (g·day^{-1})	Customary diet	43.2 ± 10.5	37.3 ± 11.2
	Ketogenic diet	2.3 ± 0.9*	2.0 ± 1.1*
Ca (mg·day^{-1})	Customary diet	810 ± 387	866 ± 406
	Ketogenic diet	1067 ± 232	1294 ± 450*
Fe (mg·day^{-1})	Customary diet	13.4 ± 2.2	13.8 ± 1.8
	Ketogenic diet	14.1 ± 2.8	15.8 ± 4.9
Na (mg·day^{-1})	Customary diet	1646 ± 556	1457 ± 873
	Ketogenic diet	1796 ± 539	2118 ± 826
K (mg·day^{-1})	Customary diet	3297 ± 1300	3678 ± 1311
	Ketogenic diet	2883 ± 536	2959 ± 518
P (mg·day^{-1})	Customary diet	1248 ± 408	1674 ± 425
	Ketogenic diet	1769 ± 539*	1981 ± 383
Mg (mg·day^{-1})	Customary diet	345 ± 93	435 ± 187
	Ketogenic diet	245 ± 49*	249 ± 58*
Zn (mg·day^{-1})	Customary diet	9.4 ± 1.9	10.7 ± 2.1
	Ketogenic diet	14.2 ± 2.9*	15.8 ± 3.5*
Vitamin A (µg·day^{-1})	Customary diet	1631 ± 863	1386 ± 1047
	Ketogenic diet	2268 ± 716	2486 ± 745*
Thiamine (mg·day^{-1})	Customary diet	0.6 ± 0.2	0.9 ± 0.4
	Ketogenic diet	1.6 ± 0.7*	1.5 ± 0.6*
Riboflavin (mg·day^{-1})	Customary diet	1.2 ± 0.4	1.4 ± 0.6
	Ketogenic diet	2.5 ± 0.4**	3.1 ± 0.7*
Vitamin C (mg·day^{-1})	Customary diet	110 ± 27	207 ± 352
	Ketogenic diet	178 ± 75*	229 ± 86
Vitamin D (µg·day^{-1})	Customary diet	9.3 ± 4.2	9.7 ± 6.0
	Ketogenic diet	17.6 ± 9.5*	17.6 ± 12.3
Vitamin E (mg·day^{-1})	Customary diet	16.8 ± 13.2	20.4 ± 12.5
	Ketogenic diet	29.5 ± 13.6*	29.6 ± 12.2
Niacin (mg·day^{-1})	Customary diet	23.5 ± 12.1	27.3 ± 10.5
	Ketogenic diet	23.0 ± 8.7	31.8 ± 9.3
Pyridoxine (mg·day^{-1})	Customary diet	2.6 ± 0.5	2.8 ± 1.2
	Ketogenic diet	2.4 ± 0.9	2.7 ± 0.9
Folate (µg·day^{-1})	Customary diet	252 ± 83	249 ± 106
	Ketogenic diet	637 ± 200*	628 ± 117*
Cyanocobalamin (µg·day^{-1})	Customary diet	0.7 ± 0.2	0.7 ± 0.2
	Ketogenic diet	11.7 ± 6.8*	11.9 ± 3.0*

Values are expressed as means ± SD. * significantly different from customary diet ($p < 0.05$)
