# Supplementary material for: Is a Four-Week Ketogenic Diet an Effective Nutritional Strategy in CrossFit-Trained Female and Male Athletes?
Source: Nutrients. 2021 Mar 6;13(3):864. doi: 10.3390/nu13030864 (PMC8001376; doi:10.3390/nu13030864)
Supplement: Supplementary file 1 [file nutrients-13-00864-s001.zip › Supplementary Table S2.pdf]

Supplementary Table S2. The Fight Gone Bad (FGB) test results.

**Indicator** Diet type	**Wall Ball Shots**	**Sumo Deadlift High Pulls**	**Box jumps**	**Press Push**	**Rowing**	**FGB$_{\text{Rounds total}}$**
			**All**			
**Round 1**						
Customary diet	26.3 ± 5.7	22.0 ± 4.9	16.7 ± 3.2	17.3 ± 3.8	12.6 ± 1.7	94.9 ± 14.3
Ketogenic diet	25.0 ± 5.4	22.5 ± 4.1	17.5 ± 2.5	18.8 ± 5.1	12.9 ± 2.0	96.8 ± 14.3
p	0.295**	0.508*	0.145*	0.096*	0.214**	0.450*
**Round 2**						
Customary diet	19.4 ± 4.6	18.0 ± 3.8	14.9 ± 3.2	14.9 ± 4.5	11.5 ± 2.2	78.7 ± 14.4
Ketogenic diet	19.7 ± 3.8	17.3 ± 1.9	15.9 ± 2.9	16.1 ± 4.7	11.3 ± 1.8	80.3 ± 11.1
p	0.530**	0.342*	**0.030***	0.095*	0.452*	0.491*
**Round 3**						
Customary diet	17.7 ± 5.0	15.9 ± 2.8	14.4 ± 3.4	14.1 ± 4.8	11.6 ± 2.9	73.7 ± 15.7
Ketogenic diet	16.7 ± 4.4	15.9 ± 3.0	14.8 ± 3.0	14.5 ± 4.3	11.8 ± 2.2	73.7 ± 13.5
p	0.175*	1.000*	0.415**	0.565*	0.655*	1.000*
**Rounds 1–3$_{\text{total}}$**						
Customary diet	63.4 ± 13.8	55.9 ± 9.5	46.0 ± 9.0	46.3 ± 12.6	35.7 ± 5.8	-
Ketogenic diet	61.4 ± 12.4	55.7 ± 7.7	48.2 ± 7.9	49.5 ± 13.6	36.0 ± 5.0	-
p	0.532**	0.875*	0.070*	0.133*	0.807**	-
			**Females**			
**Round 1**						
Customary diet	25.9 ± 6.8	22.5 ± 5.6	16.9 ± 3.8	17.5 ± 4.0	11.8 ± 1.2	94.6 ± 16.7
Ketogenic diet	24.9 ± 6.2	23.2 ± 4.8	18.1 ± 2.8	19.6 ± 5.6	12.1 ± 1.5	97.9 ± 16.8
p	0.575**	0.546*	0.140*	0.054*	0.345**	0.329*
**Round 2**						
Customary diet	18.7 ± 4.9	18.4 ± 3.7	15.1 ± 3.5	15.6 ± 3.9	10.6 ± 1.6	78.4 ± 14.0
Ketogenic diet	19.8 ± 4.5	17.6 ± 1.8	16.6 ± 3.0	16.8 ± 4.9	10.6 ± 1.7	81.4 ± 12.2
p	0.236**	0.423*	**0.022***	0.066**	1.000*	0.184*
**Round 3**						
Customary diet	17.1 ± 5.0	16.7 ± 2.3	14.5 ± 3.4	15.0 ± 4.3	11.2 ± 1.8	74.5 ± 14.1
Ketogenic diet	16.9 ± 5.2	16.8 ± 2.5	15.7 ± 2.5	15.1 ± 4.4	11.7 ± 1.7	76.2 ± 13.4
p	0.780*	0.944**	**0.042****	0.912*	0.441**	0.395*
**Rounds 1–3$_{\text{total}}$**						
Customary diet	61.7 ± 15.6	57.6 ± 9.0	46.5 ± 9.9	48.1 ± 11.6	33.5 ± 4.5	-
Ketogenic diet	61.6 ± 14.9	57.6 ± 7.8	50.4 ± 7.8	51.5 ± 14.3	34.4 ± 4.3	-
p	1.000**	1.000*	**0.014***	0.110**	0.373*	-
			**Males**			
**Round 1**						
Customary diet	27.0 ± 2.9	21.0 ± 3.0	16.4 ± 1.7	17.0 ± 4.0	14.2 ± 1.3	95.6 ± 9.2
Ketogenic diet	25.2 ± 4.0	21.2 ± 1.6	16.4 ± 1.3	17.2 ± 4.1	14.6 ± 1.9	94.6 ± 8.4
p	0.285**	1.000**	1.000**	0.686**	0.423**	1.000**
**Round 2**						
Customary diet	20.8 ± 4.0	17.2 ± 4.3	14.4 ± 2.9	13.6 ± 5.7	13.4 ± 2.1	79.4 ± 17.0
Ketogenic diet	19.6 ± 2.1	16.6 ± 2.1	14.4 ± 2.4	14.8 ± 4.3	12.6 ± 1.1	78.0 ± 9.5
p	0.686**	0.686**	1.000**	0.787**	0.361**	0.787**
**Round 3**						
Customary diet	19.0 ± 5.2	14.4 ± 3.5	14.2 ± 3.8	12.2 ± 5.5	12.4 ± 4.7	72.2 ± 20.4
Ketogenic diet	16.2 ± 2.4	14.2 ± 3.3	13.0 ± 3.4	13.4 ± 4.3	12.0 ± 3.2	68.8 ± 13.8
p	0.144**	0.655**	0.180**	0.500**	0.593**	0.225**
**Rounds 1–3$_{\text{total}}$**						
Customary diet	66.8 ± 9.8	52.6 ± 10.5	45.0 ± 8.0	42.8 ± 15.0	40.0 ± 6.3	-
Ketogenic diet	61.0 ± 6.0	52.0 ± 6.7	43.8 ± 6.6	45.4 ± 12.3	39.2 ± 5.3	-
p	0.418**	0.787**	0.465**	0.715**	0.423**	-

Note: Values are expressed as the mean ± standard deviation. * Data were analysed by repeated-measured one-way analysis of variance. ** Data were analysed by the Wilcoxon signed-rank test. Statistical significance was set at $p < 0.05$.
